# Tumor cell senescence response produces aggressive variants

**DOI:** 10.1038/cddiscovery.2017.49

**Published:** 2017-08-21

**Authors:** Leixiang Yang, Jia Fang, Jiandong Chen

**Affiliations:** 1 Molecular Oncology Department, H. Lee Moffitt Cancer Center, 12902 Magnolia Drive, Tampa, FL 33612, USA; 2 Tumor Biology Department, H. Lee Moffitt Cancer Center, 12902 Magnolia Drive, Tampa, FL, USA

## Abstract

Tumors often respond favorably to initial chemotherapy but eventually relapse with drug resistance and increased metastatic potential. Cellular senescence is a major therapeutic outcome of cancer chemotherapy, which leads to tumor stasis or regression through immune clearance of senescent cells. However, senescent tumor cells have been shown to resume proliferation at low frequency. We found that subjecting arrested senescent tumor cells to cytotoxic treatments stimulates the clonogenic proliferation of remaining survivors. The senescence revertants showed a reduced rate of proliferation but increased migration and invasion potential *in vitro*, and increased tumorigenic potential *in vivo*. Gene expression profiling showed that the senescence revertants are distinct from both parental and senescent cells. A subset of senescence-activated genes remains active in the revertants. These genes are implicated in regulating cell motility, invasion, and metastasis, which may collectively contribute to the aggressiveness of the revertants. The findings suggest that although therapy-induced senescence has short-term benefits, the response also causes reprogramming of gene expression and activates invasion-related genes that accelerate tumor progression.

## Introduction

Post-treatment relapse and distant metastasis are major causes of mortality by solid tumors. There is extensive preclinical evidence in animal models that nearly all types of cancer treatments, from surgery to chemotherapy and targeted therapy, can stimulate tumor spread.^1^ Modification of the host microenvironment by the treatments plays an important role by providing survival niches for metastatic tumor cells. Furthermore, tumor cells are genetically unstable. Induction and subsequent Darwinian evolution of drug-resistant cells play important roles in therapy-induced progression. It is likely that additional mechanisms also contribute to this phenomenon.

Replicative senescence was initially discovered as a cell culture phenomenon. Excessive cell division results in telomere erosion, activation of DNA damage signaling, and irreversible arrest mediated by the pRb and p53 tumor suppressors.^2^ Senescent cells in culture exhibit large flat cell morphology, increased p21/p16 levels, active metabolism, and increased lysosomal SA-*β*-galactosidase (SA-*β*-gal) activity. Radiation and chemotherapy drugs also induce premature senescence that is similar to replicative senescence.^3^ SA-*β*-gal staining suggests that senescence occurs *in vivo* in aging tissues, premalignant lesions, and in tumors treated with chemotherapy.^4^ Mouse models showed that tumor cells with defective apoptosis pathway respond to chemotherapy by entering senescence. Senescent tumor cells secrete many pro-inflammatory cytokines, growth factors, and matrix metalloproteases (the SASP phenotype). These factors act through paracrine and autocrine mechanisms to promote immune clearance and tumor remission. Therefore, senescence is an important tumor suppression mechanism.^5^

Senescence cells undergo self-sustaining cell-cycle arrest involving stable epigenetic silencing of proliferation genes.^4^ Silenced E2F target genes form heterochromatin foci (SAHF) in some senescent cells.^6^ Senescent cells also upregulate many pro-inflammatory genes.^2^ Presumably, senescence involves establishing and maintaining positive feedback loops in the heterochromatinization of cell-cycle genes and activation of senescence-specific genes. Heterochromatin proteins such as HP1 and SUV39H1 bind to dimethylated H3K9 and then promote further methylation of adjacent H3K9. Therefore, they help maintain self-perpetuating positive feedback loops and stable repression. In addition to transcription repression, senescent cells display constitutively active DNA damage signaling.^7^ Paracrine and autocrine effects from the SASP factors also play a role in maintaining positive feedback activation of gene expression and senescence arrest.^8,9^

Tumor cells that are resistant to apoptosis often respond to chemotherapy by entering premature senescence. Normal stromal fibroblasts also enter senescence after DNA-damaging treatment. Although senescence is generally perceived as a form of irreversible cell-cycle arrest, studies of drug-induced senescence showed that senescent tumor cells in culture spontaneously revert to active proliferation at low frequency.^10^ Inactivation of p53 or pRb in early stage senescent cells is often sufficient to stimulate cell-cycle re-entry.^11^ Our recent study showed that deficiency in nucleolar rRNA transcription repression significantly increases the frequency of senescence reversal.^12^ Therefore, after termination of drug treatment, senescent tumor cells may eventually produce proliferative clones and result in relapse.

In addition to causing relapse, senescence reversal of tumor cells may have other adverse effects. Recent studies suggest that tumor cells in culture that have undergone senescence arrest re-emerge with increased levels of certain tumor stem cell markers.^13,14^ Normal human fibroblasts undergoing replicative senescence acquire DNA hypomethylation/hypermethylation patterns similar to cancer cells.^15^ Furthermore, the cancer-like DNA methylation pattern is partially retained after the senescent fibroblasts are forced to proliferate by SV40 T antigen expression.^15^ Senescent fibroblasts forced to re-enter the cell cycle by p53 inactivation retain the expression of many genes associated with senescence.^16^ Therefore, senescence in fibroblasts creates long-lasting imprints on the epigenome and certain gene expression programs. Similar reprogramming may also occur in tumor cells that have undergone senescence reversal.

Chemotherapy promotes the emergence of drug-resistant and simultaneously more malignant tumor cells.^17,18^ Induction chemotherapy has been shown to significantly accelerate the re-growth of NSCLC compared with untreated tumors.^19^ Multiple mechanisms, such as selection of pre-existing mutant clones and activation of stress-resistant genes by epigenetic mechanisms, are responsible for some of the effects. Tumor stem cells that exist in a stress-resistant epigenetic state in the population may be enriched by the chemotherapy and contribute to relapse and metastasis.^20,21^ Stromal fibroblast senescence and production of SASP factors can promote tumor cell proliferation and invasion through paracrine mechanism, creating a microenvironment for metastasis.^9^ Whether tumor cell senescence response also promotes progression is unclear.

Results described in this report show that tumor cell senescence is frequently reversed after stimulation by a variety of stress signals. Reversal from senescence produces tumor cells that are distinct from the parental cells, exhibiting altered gene expression profile and increased invasiveness. The results suggest that senescence response to DNA damage by tumor cells may contribute to the phenomenon of therapy-induced progression.

## Results

### Stress treatment of senescent tumor cells promotes cell-cycle re-entry

**Figure 1:**
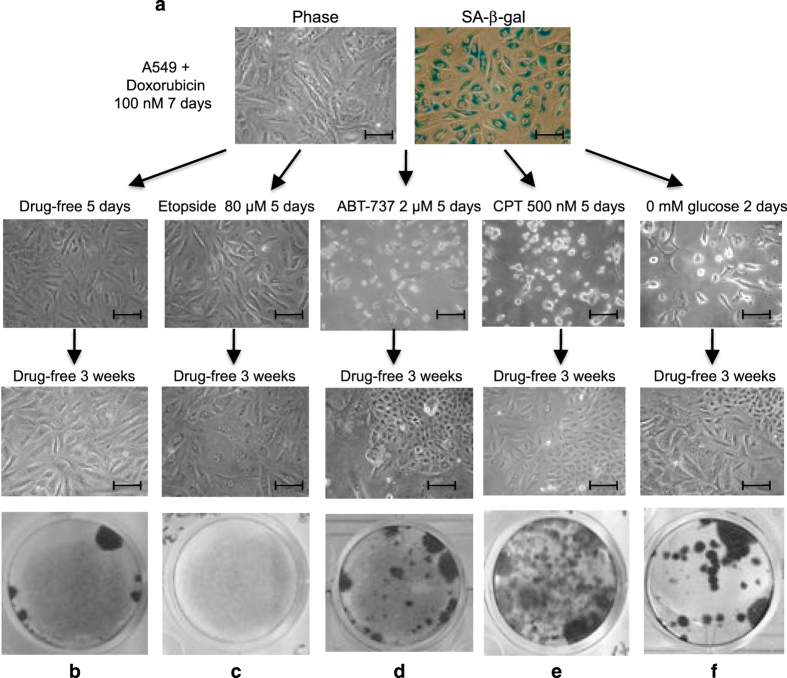
Stress treatments stimulate senescence reversal. (**a**) A549 cells were treated with 100 nM doxorubicin for 7 days and stained for SA-*β*-galactosidase activity. Cell morphology was photographed under phase-contrast microscope. Bar=20 *μ*m. (**b**–**f**) Senescent A549 cells induced by doxorubicin were mock treated (**b**) or treated with different stimuli, including 80 *μ*M etopside (**c**), 2 *μ*M ABT-737 (**d**), 500 nM camptothecin (**e**), and glucose deprivation (**f**) for indicated time. The cells were then washed and cultured in drug-free media for 3 weeks. Colonies were stained with crystal violet. Bar=20 *μ*m.

Reversal from drug-induced senescence has been implicated as a mechanism of tumor recurrence.^10^ Therefore, we were interested in identifying secondary treatments that can reduce the frequency of senescence reversal. As a cell culture model of therapy-induced senescence, A549 lung tumor cells were treated 7 days with 100 nM doxorubicin, which resulted in cell-cycle arrest and SA-*β*-gal positive staining (Figure 1a). Following drug treatment, the cells were washed and cultured in drug-free medium for 20–30 days and stained for colony formation. The treatment resulted in >90% SA-*β*-gal-positive cells and >99% of the cells remained arrested 21 days after drug removal (Figure 1b).

To test the impact of different stress signals on senescence maintenance, the senescent cells were incubated with drugs or glucose-free medium. After removing the drugs by washing, the cells were incubated in drug-free medium for 21 days to determine colony formation efficiency. The results with several drugs showed drastically different outcomes. Secondary treatment with etoposide did not induce noticeable cell death or morphological change, but significantly reduced background colony formation efficiency (Figure 1c). In contrast, the Bcl2 inhibitor ABT-737 induced significant death in senescent cells, but surviving cells formed colonies at much higher frequency than background (Figure 1d). Camptothecin (CPT) and glucose deprivation induced significant cell rounding and detachment. The small number of surviving cells also formed colonies at high efficiency in drug-free medium (Figures 1e and f).

In similar experiments, the RNA Pol I inhibitor CX5461 was used to induce senescence in A549 cells.^12,22^ Secondary treatments with ABT-737, CPT, and glucose starvation also induced partial cell death accompanied by significant colony formation (Supplementary Figure S1). These results suggest that stress treatments can stimulate senescent cells to re-enter cell cycle.

### Inhibition of Bcl2 promotes senescence escape

**Figure 2:**
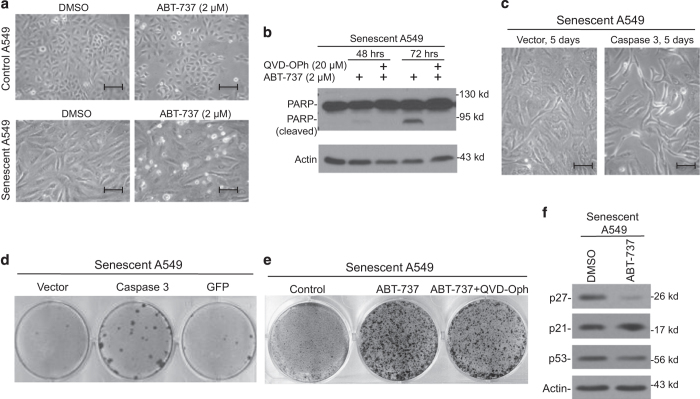
Apoptosis stimulates senescence reversal. (**a**) Senescent A549 cells were treated with 2 *μ*M Bcl2 inhibitor ABT-737 for 2 days and photographed to show apoptotic cell death. Bar=20 *μ*m. (**b**) Senescent A549 cells induced by doxorubicin were treated with 2 *μ*M ABT-737 in the presence of pan-specific caspase inhibitor QVD-OPh (20 *μ*M) for indicated times. The cells were analyzed for the indicated markers by western blot. (**c**) Senescent A549 cells were transfected with pCMV-caspase-3. Cell morphology was photographed 5 days after the transfection. Bar=20 *μ*m. (**d**) Senescent A549 cells were transfected with the indicated plasmids and cultured for 7 weeks. Colony formation was detected by crystal violet staining. (**e**) Senescent A549 cells were treated with 2 *μ*M ABT-737 in the presence or absence of 20 *μ*M QVD-OPh for 3 days, the cells were washed and cultured in drug-free medium until colonies formed. Colonies were stained with crystal violet. (**f**) Senescent A549 cells were treated with DMSO control or 2 *μ*M ABT-737 for 3 days. The expressions of cell-cycle proteins in senescent cells after removing the stimuli for 24 h were analyzed by western blot.

The ability of Bcl2 inhibitor ABT-737 to induce clonogenic growth of senescent cells was of interest because a derivative of this compound is used in clinical trials. Non-senescent A549 cells were not sensitive to apoptosis by ABT-737 (Figure 2a). However, senescent cells showed significant sensitivity to ABT-737-induced apoptosis and PARP cleavage (Figures 2a and b), consistent with recent report using senescent fibroblasts.^23^ Transient knockdown of Bcl2 using siRNA also induced colony formation in senescent A549 cells (Supplementary Figures S2a–d). Senescent H460 cells were also induced to proliferate by ABT-737 treatment (Supplementary Figure S2e), suggesting that the phenomenon is not restricted to A549 cells alone. To directly test whether activation of apoptotic caspase stimulates senescence reversal, senescent A549 cells were transiently transfected with Caspase 3, followed by colony formation analysis. Transfection of Caspase 3 induced partial cell death (Figure 2c). Following long-term culture, significant colony formation was observed (Figure 2d). This result suggests that direct activation of caspase 3 increases the probability of senescence escape.

To test whether ABT-737-mediated senescence reversal requires caspase activation, cells were treated with combination of ABT-737 and broad spectrum caspase inhibitor QVD-OPh. QVD-OPh completely blocked PARP cleavage and apoptosis induced by ABT-737 (Figure 2b) but only moderately reduced colony formation efficiency (Figure 2e), suggesting that both caspase-dependent and -independent mechanisms were involved. The cell death caused by CPT was not blocked by the caspase inhibitor QVD-OPh (Supplementary Figure S3a), suggesting that it was not apoptosis. Staining of the cells treated with CPT or glucose starvation with fluorescent dyes Hoechst 33342 and propidium iodide revealed that cells mainly undergo necrosis (Supplementary Figure S3b). The results above suggest that senescence arrest in tumor cells is susceptible to disruption by various stress signals. Necrosis is a clinical relevant form of stress *in vivo* since large tumors often contain necrotic regions due to poor blood supply.

Analysis of cell-cycle markers revealed that ABT-737 reduced p27 levels, while the level of p53 and p21 were not significantly affected (Figure 2f). The expression of p27 in proliferating A549 cells was not affected by ABT-737 (data not shown). Bcl2 has been shown to promote p27 expression, cell-cycle arrest, and senescence.^24,25^ ABT-737 may stimulate the proliferation of senescent cells partly by inhibiting Bcl2 and downregulating p27.

### Senescence revertants exhibit increased migration and invasion

**Figure 3:**
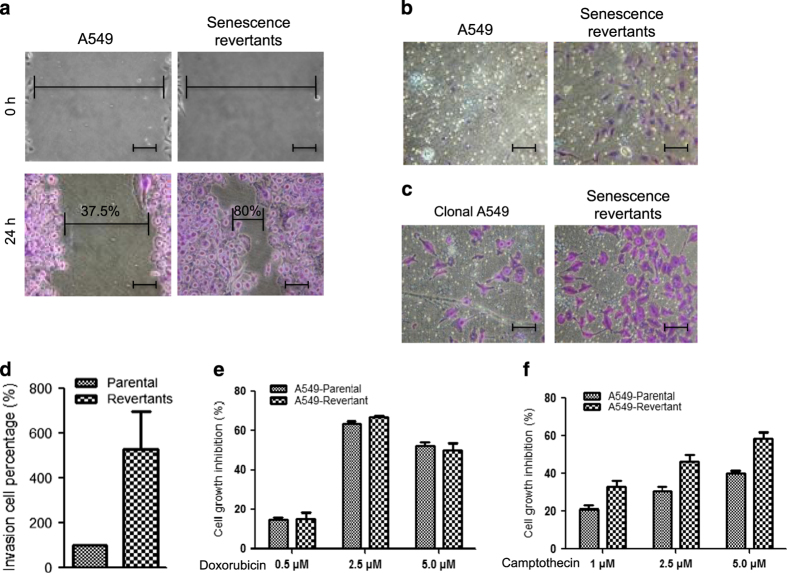
Senescence revertants have increased migration and invasion potential. (**a**) Cell migration activity of A549 parental cells and senescence revertants was compared using wound healing assay. Upper panels show the identical gaps created at the beginning of the assay. Cells were cultured for 24 h and stained with crystal violet to determine the closure percentage. Bar=20 *μ*m. (**b **and **c**) Glucose starvation-induced escape colonies from senescent A549 were cultured in Matrigel chambers; cell transition through the matrix was stained after 48 h. Bar=20 *μ*m. (**d**) Quantitation of (**b**). Values are mean±S.D. of triplicates. (**e** and **f**) A549 doxorubicin-induced senescence revertants were treated with doxorubicin or camptothecin for 3 days; cell death was determined by MTT assay.

Senescent cells undergo extensive epigenetic and gene expression changes, which may not be completely erased when cells resume proliferation.^15^ To test whether the senescence revertants gained aggressive features, the revertant colonies were pooled and analyzed in wound healing assay and Matrigel invasion assay. The results revealed that starvation-induced revertants showed stronger migration (Figure 3a) and increased ability to traverse Matrigel barrier (Figures 3b and d). Spontaneous revertants also showed similar results (not shown), suggesting that senescence revertants are inherently more invasive irrespective of whether they arise spontaneously or induced by stress treatments.

Since initial experiments were performed using A549 cells with unknown passage number, the senescence/reversal procedure may enrich a pre-existing subpopulation. Therefore, we derived a clonal A549 cell line from a single cell and repeated the experiments. Similar increase of invasiveness was observed from the senescence revertants (Figure 3c). Furthermore, the revertants showed no increase in resistance to different chemotherapy drugs in short-term survival test (Figures 3e and f), arguing against the selection of cancer stem cells or drug-resistant cells in our assay. Instead, the results suggested that the process of entering and exiting senescence produced reprogrammed aggressive cells.

### Persistent activation of a subset of senescence genes in revertants

**Figure 4:**
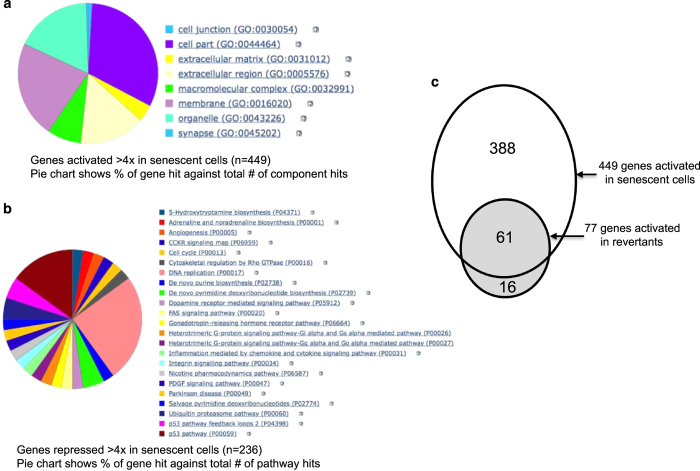
Senescence revertants retain activation of a subset of senescence genes. (**a**) Cellular component of genes activated in senescent cells based on PANTHER gene ontology analysis. (**b**) Pathways of genes downregulated in senescent cells based on PANTHER gene ontology analysis. (**c**) Overlap of genes activated in senescent cells and senescence revertants. Sixty-one of 77 genes activated in senescence revertants were also activated in senescence cells.

RT-PCR analysis using a focused cancer stem cell gene panel did not reveal increased expression of stem cell markers in the senescence revertants (Supplementary Figure S4). To further investigate the basis of increased invasiveness in the senescence revertants, RNA-seq was performed on proliferating A549, arrested senescent cells, and proliferating revertants (all derived from the same clonal A549 cell line). SA-*β*-gal staining confirmed there were no senescent cells in the proliferating revertant culture. Senescent A549 cells showed strong activation of large numbers of genes (449 genes activated >4-fold; Supplementary Table S1). PANTHER gene ontology analysis showed that 41% of the activated genes encode proteins that were membrane-associated, extracellular matrix, or secreted proteins (Figure 4a).^26^ DAVID analysis also suggested strong functional clustering in extracellular and immunity functions.^27^ There were 236 genes downregulated by >4-fold in senescent cells (Supplementary Table S2); 25% of these genes were involved in DNA replication (Figure 4b). DAVID analysis of the downregulated genes also suggested strong functional clustering in cell cycle and cell division. The results were consistent with the activation of pro-inflammatory pathways and stable cell-cycle arrest in senescent cells.

The revertants had 77 genes activated >2 fold compared with A549 (*P*<0.1), these genes were highly reproducible by RT-PCR analysis (35/39, see below). Interestingly, 61 (79%) of these genes were also activated >2-fold in senescent cells (Figure 4c and Supplementary Table S3). There were 63 genes repressed >2-fold in the revertants relative to A549 (*P*<0.1; Supplementary Table S4). Unlike the activated genes, the repressed genes in the revertants did not overlap with repressed genes in senescent cells, showed no clear functional clustering in DAVID analysis, and had poor confirmation rate by RT-PCR (0/7). Therefore they were not investigated further. The high degree of overlap in activated genes (Figure 4c) suggests that the revertants were originated from senescent cells and retained activation of a subset of senescence genes. Furthermore, there was no strong activation of other genes in the revertants that would suggest the selection of a unique population such as stem cells or drug-resistant cells.

### Validation of revertant genes

To validate the RNA-seq results, 39 genes that were activated in both senescent cells and revertants were selected for analysis by RT-PCR. Activation was confirmed in both senescent cells (39/39, 100%) and in revertants (35/39, 90%; Table 1). In contrast, seven of seven repressed genes failed confirmation by RT-PCR (not shown).

To test the cell type specificity of senescence-mediated long-term gene activation, 19 A549 genes were selected for analysis in senescence revertants derived from H1299 and MCF7 cells. The results showed strong similarity between NSCLC cell lines A549 and H1299. Although some A549 genes (9/19) were not expressed in H1299, between the expressed genes senescent A549 and H1299 cells shared 100% similarity (10/10), and the revertants shared 80% similarity (8/10) (Table 2). In contrast, the similarity between A549 and MCF7 (breast tumor) was significantly weaker. Although most A549 genes were also expressed in MCF7 (18/19) and senescent A549 and MCF7 shared 72% (13/18) activated genes, the magnitude of activation in MCF7 was significantly lower. Furthermore, there was only 7% (3/18) similarity between A549 and MCF7 revertants (Table 2). The results suggest that cells of the same tumor type shared significant similarity in gene activation during senescence and reversal. There is significant cell type specificity in the genes that remain activated after senescence reversal.

In gene ontology analysis, the A549 revertant-activated genes also showed significant bias towards membrane-associated or extracellular proteins as expected from their significant overlap (79%) with senescence-activated A549 genes. Further literature search of these genes revealed that most were linked to cell adhesion, migration, and metastasis, suggesting that they may be responsible for the enhanced invasiveness in senescence revertants (Table 2). In general, the magnitude of activation of the revertant genes was significantly smaller compared with their expression level in senescent cells (e.g., NPTX1 was activated 40-fold in senescent cells *versus* 6-fold in revertants; Table 1).

### Senescence reversal as a potential source of non-genetic heterogeneity

**Figure 5:**
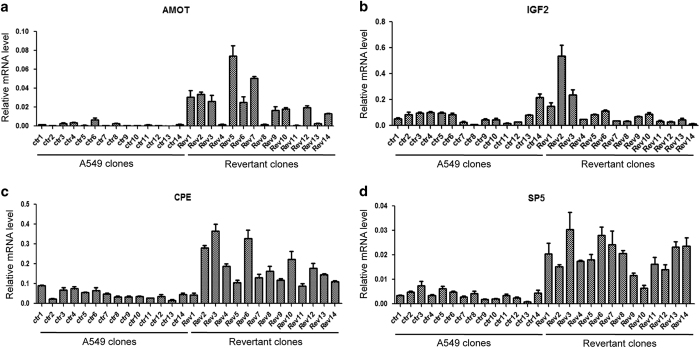
Gene expression heterogeneity in senescence revertant clones. A clonal A549 cell line was used to generate 14 clonal sublines by limited dilution. The same cell line was also treated with doxorubicin to generate senescence revertant colonies. Fourteen revertant colonies were cloned and expanded into cell lines. The expression of AMOT (**a**), IGF2 (**b**), CPE (**c**) and SP5 (**d**) in 14 A549 clonal sublines and 14 clonal revertant cell lines were analyzed by RT-qPCR. Values are mean±S.D. for experimental triplicates.

The RNA-seq analysis and RT-PCR validation was performed using pooled revertant colonies. To determine whether there was clonal variability of gene activation in the revertants, a clonal A549 was used to generate senescence revertants and clonal cell lines were established from individual revertant colonies. As control, the parental clonal A549 was also used to establish single-cell subclones. RT-PCR analysis of several genes from Table 1 suggested clonal variation in the expression of AMOT and IGF2 in the revertants (Figures 5a and b), whereas the same revertant clones showed relatively uniform increase of CPE and SP5 expression (Figures 5c and d). Therefore, senescence reversal may expand the range of expression level of a subset of genes, producing subclones with different combination of activated genes.

### Senescence revertants have reduced proliferation rate *in vitro*

**Figure 6:**
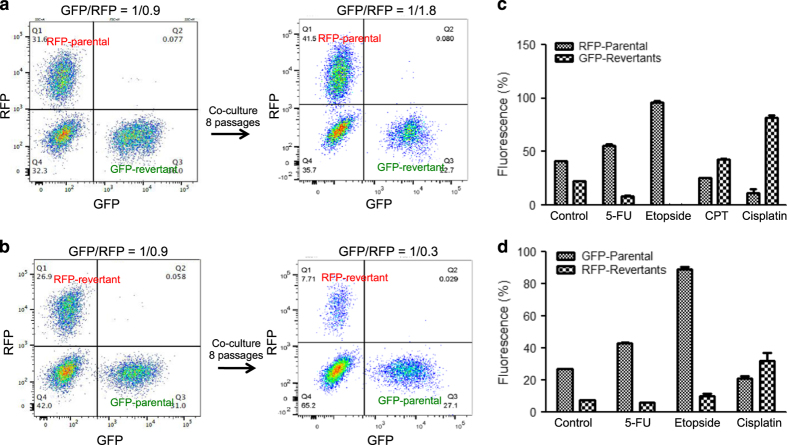
Senescence revertants have reduced proliferation potential *in vitro*. (**a**) A549 cells stably infected with lentivirus expressing GFP or RFP were used to generate senescence revertants from doxorubicin treatment. The RFP-labeled parental cells and GFP-labeled revertants were mixed at 1 : 1 ratio, co-cultured for eight passages, and analyzed by FACS. (**b**) GFP-labeled parental cells and RFP-labeled revertants were mixed at 1 : 1 ratio, co-cultured for 8 passages, and analyzed by FACS. (**c** and **d**) RFP and GFP-labeled cells were mixed 1 : 1 and treated with 50 *μ*M 5-FU, 40 *μ*M etoposide, 0.5 *μ*M CPT and 20 *μ*M cisplatin. The cells were treated for 7 days, followed by 14-day culture in drug-free medium. The treatment was repeated once and the surviving cells were analyzed by FACS.

During routine culture, the pooled senescence revertants did not show noticeable difference in proliferation rate compared with parental cells. To detect more subtle changes, the clonal A549 cells and pooled senescence revertants were labeled with lentivirus expressing H2B-RFP and H2B-GFP respectively.^28^ The labeled cells were mixed at 1 : 1 ratio and cultured for eight passages. FACS analysis of RFP/GFP ratio showed that parental cells in the mixture out grew the senescence revertants during co-culture, irrespective of the fluorescence marker (Figures 6a and b). Therefore, although the revertants were more motile and invasive, they had a slight proliferative disadvantage compared with parental cells under normal culture conditions. When the fluorescent-labeled cells were co-cultured in the presence chemotherapy drugs for several passages, the revertants showed a growth advantage in the presence of cisplatin and CPT, whereas 5-FU and etoposide had no effect or even preferentially blocked the growth of revertants (Figures 6c and d). The results suggest that under certain stress conditions, the senescence revertants have a competitive advantage over the parental cells.

### *In vivo* competition between revertant and parental cells

**Figure 7:**
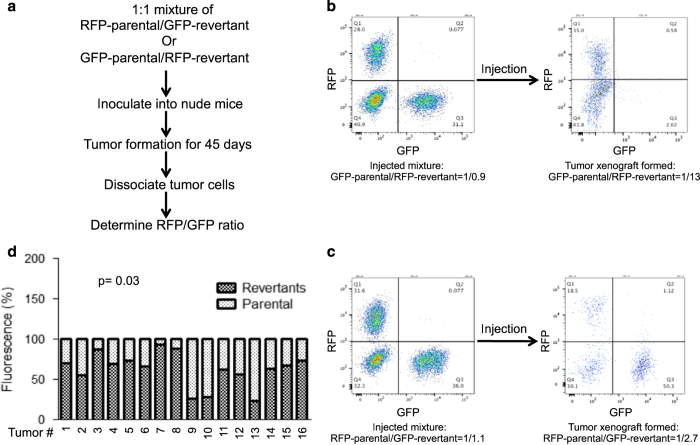
Senescence revertants exhibit growth advantage *in vivo*. (**a**) A schematic diagram of the *in vivo* competition assay. (**b **and **c**) GFP/RFP-labeled parental A549 and senescence revertants were inoculated subcutaneously into nude mice. After the tumor nodules were formed, the tumors cells were isolated and analyzed for the GFP/RFP ratio by FACS. The ratios of cell mixtures before injection and from representative tumors are shown. (**d**) Summary of parental/revertant cell ratios in 16 tumors (8 from RFP-parental/GFP-revertant mixture and 8 from GFP-parental/RFP-revertant mixture).

To determine whether the senescence revertants can compete with parental cells under *in vivo* growth condition, 1 : 1 mixture of GFP-parental:RFP-revertant and RFP-parental:GFP-revertant cells were inoculated into nude mice subcutaneously (Figure 7a). The tumors formed by the cell mixture were dissociated into single cells and analyzed by FACS to determine the GFP/RFP ratio (Figures 7b and c). The results showed that in 13/16 tumors the revertant cells outgrew the parental cells (*P*=0.03; Figure 7d). Therefore, although the senescence revertants had a proliferative disadvantage in culture, they were able to out-compete the parental cells under more stressful *in vivo* tumor growth condition. We also inoculated the cell mixture through tail vein injection in an attempt to compare metastasis with the lung. However, the analysis was uninformative due to the low percentage of fluorescent tumor cells in the lung cell mixture (not shown).

## Discussion

DNA-damaging chemotherapy induces senescence in both stromal and tumor cells. Senescent stromal fibroblasts have been shown to promote tumor growth and metastasis through secretion of SASP factors. The results described in this study suggest that senescence response by tumor cells may also contribute to therapy-induced progression by producing invasive revertants. The basis of this phenomenon is the ability of senescent tumor cells to re-enter the cell cycle when disturbed by various forms of stress, and the stable activation of a subset of genes implicated in cell motility and metastasis in the revertants.

Contrary to the common perception that senescence is an irreversible arrest, previous work showed that inactivation of p53 in human fibroblasts undergoing replicative senescence can lead to cell-cycle re-entry.^29^ Therefore, after cells have committed to senescence, disruption of cell-cycle regulators can cause reversion to active proliferation. Spontaneous escape from drug-induced senescence arrest has been suggested to be a mechanism of tumor relapse.^10^ Our current results showed that several cytotoxic treatments that induce apoptosis and necrosis can stimulate senescent tumor cells to re-enter cell cycle. This cell culture phenomenon is likely to be clinically relevant, since senescent cells in tumors are exposed to a variety of stresses due to chemotherapy treatments, hypoxia and nutrient deprivation.

Although the mechanism of senescence reversal by these treatments remains to be investigated in detail, previous studies showed that apoptotic cells activate neighboring cells through secretion of prostaglandin E2.^30,31^ Caspase 3 activation leads to cleavage of pRb, which is an important mediator of senescence.^32^ In addition to pRb, Caspase 3 cleaves a large number of proteins involved in chromatin modification.^33^ It is possible that lethal levels of Caspase 3 stimulates neighboring cells through paracrine mechanisms, whereas sublethal Caspase 3 activation causes changes in heterochromatin in senescent cells, increasing the probability of reactivating genes essential for cell cycle.

Classic drug resistance selection scheme using cytotoxic levels of drugs can enrich for survivors with cancer stem cell features.^14,20,21^ Previous studies suggested that tumor cells spontaneously reverted from drug-induced senescence were more stem-like.^13,14^ Our results suggest that the non-lethal levels of DNA damage in our experiments did not select for or induce stem cells, but instead induced stable changes in gene expression through activating the senescence response program. Establishing a senescence state involves downregulation of large number of genes involved in DNA replication and cell division, and activation of numerous genes in inflammation, motility, invasion, and cell–cell communication. The return to active cell cycle requires restoring normal expression of DNA replication and cell division genes. However, a subset (~10%) of senescence-activated genes remains activated. The degree of activation of these ‘revertant genes’ were generally weaker than in senescent cells, suggesting that the upstream signals that regulate their expression in senescent cells were decreased or absent in the revertants.

It is possible that epigenetic changes and positive feedback loops become self-perpetuating for a subset of senescence genes, resulting in continued activation without upstream signals in the revertants. Our comparison of lung and breast tumor cell lines suggests that different cell types may retain expression of distinct subset of senescence genes after reversal. Previous study revealed strong cell type-specific difference in gene expression profile when comparing the senescent state.^34^ The difference in chromatin state and tissue-specific gene expression may dictate what genes are activated during senescence, and remain active after re-entering the cell cycle. Interestingly, the revertants have a growth disadvantage compared with parental cells under normal cell culture condition, suggesting that there may be a tradeoff in proliferation rate for the continued expression of some senescence genes. However, in a tumor xenograft environment, the revertants were able to out-compete the parental cells, suggesting that proliferation rate alone does not determine tumorigenic potential *in vivo*.

Tumor cell heterogeneity presents a significant hurdle to curative treatment.^35^ Genetic and epigenetic heterogeneity facilitates Darwinian evolution, and predicts tumor progression.^36,37^ Non-genetic transient or semi-permanent gene expression changes also promote treatment resistance and progression.^20^ Understanding the cause of tumor cell heterogeneity is necessary for developing treatments that prevent its emergence. Our results suggest that senescence response by tumor cells may provide a non-genetic mechanism of increasing tumor heterogeneity, since a subset of the revertant genes were activated with significant degree of clonal variability. It is possible that in the absence of sustained upstream signaling, the revertant genes were turned off in a random fashion, producing clones with different combination of gene activation and heterogeneity.

Chemotherapy is often given in cycles with significant gaps in between to allow for recovery from side effects. The results of our study suggest that apoptotic or cytotoxic agents may have unintended activities that cause activation of senescent tumor cells from prior treatments, generating new variants with stable activation of genes that stimulate invasion and metastasis. Multiple cycles of senescence and reversal may further increase the number of activated genes and increase the level of clonal heterogeneity. This phenomenon underscores the complexities of cancer therapy and may contribute to the lack of overall survival benefit of certain treatments despite favorable short-term responses. The results suggest that blocking the initiation of senescence, or inhibiting reversal from senescence, may reduce the chance of tumor progression.

## Materials and methods

### Cell lines and reagents

The human NSCLC cell lines A549, H460, H1299 and human breast cancer cell line MCF7 were purchased from the ATCC (Manassas, VA, USA) from 1995 to 2000 and maintained in Dulbecco modified Eagle medium (DMEM) with 10% fetal bovine serum. Low passage (<5) original stocks were used for the experiments. The cells have not been reauthenticated, but were routinely monitored for morphological characteristics and p53 status by western blot (wild-type p53 for A549/H460/MCF7, p53-null for H1299). MCF7 was also monitored for p53 codon 72 polymorphism by western blot. Cells were cultured at a 37 °C incubator in a humidified atmosphere containing 5% CO_2_. A549 cells with stable expression of GFP and RFP were generated by infecting A549 cells with LV-GFP and LV-RFP viruses. The LV-GFP and LV-RFP plasmids were gifts from Elaine Fuchs (Addgene plasmid # 25999 and # 26001). Corning Biocoat Matrigel Invasion Chambers was obtained from Corning (Bedford, MA, USA). Mouse anti-PARP, mouse anti-p53 (DO1), mouse anti-p27, and mouse anti-p21 were purchased from BD Biosciences (San Jose, CA, USA). Mouse anti-*β*-actin was obtained from Sigma (St Louis, MO, USA). Rabbit anti-Bcl2 antibody was purchased from Cell Signaling Technology (Danvers, MA, USA). MTT (3-(4,5-dimethylthiazol-2-yl)-2,5-diphenyltetrazolium bromide) was obtained from Sigma. Human cancer stem cell PCR array (PAHS-176ZA) was purchased from Qiagen (Germantown, MD, USA). Data processing was performed using the manufacturer website.

### SA-*β*-galactosidase staining

A549 cells (3×10^4^/well) were seeded to 24-well plates for overnight before being treated with 100 nM doxorubicin. After 7 days of the treatment, SA-*β*-galactosidase staining was carried out using the Senescence histochemical staining kit (Sigma) following the manufacturer's instruction. Cells were incubated at 37 °C for 18 h and photographed.

### Colony formation assay

A549 cell senescence was induced by the treatment with 100 nM doxorubicin for 7 days. The cells were washed twice with PBS to remove doxorubicin. The senescent cells were later treated with different drugs, including 80 *μ*M etopside, 500 nM camptothecin, 2 *μ*M ABT-737, cultured in glucose-free medium for 5 days, or transfected with 2 *μ*g of Caspase 3 plasmid for 24 h. After removing the stimuli, cells were cultured in medium for 3–7 weeks with re-feeding every 5 days until colonies were visible. Colonies were stained by 0.5% crystal violet.

### RNA interference (RNAi)

Senescence cells induced by 0.1 *μ*M doxorubicin for 7 days were transfected with 50 nM control siRNA ( AATTCTCCGAACGTGTCACGT), Bcl2 siRNA1 ( GGATGACTGAGTACCTGAA), and Bcl2 siRNA2 ( GGAGAACAGGGTACGATAA) (Invitrogen, Carlsbad, CA, USA) using RNAiMAX (Invitrogen) according to the instructions from the supplier. After 48 h of transfection, cells were cultured in normal DMEM medium to observe colonies formation. The knock down efficiency of Bcl2 was detected both on mRNA and protein level.

### Cell growth inhibition assay

A549 parental and revertant cells (5×10^3^/well) were seeded to 96-well plate. After 24 h incubation, cells were treated with different concentrations of doxorubicin or camptothecin for 72 h. Cell growth inhibition was detected by MTT assay. Briefly, 10 *μ*l MTT (final concentration 5 mg/ml) was added to each well and after 3 h incubation in cell culture incubator, the culture medium was removed and 200 *μ*l DMSO was added to each well to dissolve the formazan. The optical density was measured spectrophotometrically at 570 nm.

### Wound healing assay

*In vitro* wound healing assay was used to measure the migration ability of A549 parental cells or A549 cells escaped from senescence. The cells (4×10^5^/well) were plated in six-cell plates for 18 h to form a confluent monolayer. The cells were then maintained in serum-free medium for 18 h before scraping in a straight line with a P200 pipette tip. After removing the debris, cells were maintained in regular medium for 24–48 h, stained with 0.5% crystal violet, and photographed under a phase-contrast microscope.

### Transwell assay

The Matrigel invasion chamber (Corning) was used to assess the invasive property of A549 parental cells or A549 revertants *in vitro*. The assay was based on the manufacturer protocol. The cells were cultured in serum-free medium for 18 h, trypsinized, and suspended in serum-free medium at 5×10^4^ cells/ml for 24-well chambers. The chamber was then inserted to the wells containing chemoattractant and cultured in the humidified cell culture incubator for 24–48 h. Cell invasion was measured by crystal violet staining and photographed.

### Western blot

After the indicated treatments, cells were lysed in lysis buffer (50 mM Tris-HCl, pH 8.0, 150 mM NaCl, 0.5% NP-40, 1 mM phenylmethylsulfonyl fluoride, protease inhibitor cocktail) and centrifuged for 10 min at 14 000×*g* to remove insoluble debris. The supernatant was boiled in sample buffer for 5 min and subjected to SDS-PAGE and western blot to detect the expression of indicated proteins using their corresponding antibodies.

### Necrosis assay

A549 cell senescence was induced by 7 days treatment with 100 nM doxorubicin. The cells were then treated with 500 nM camptothecin or cultured in glucose-free medium for 48 h. Hoechst 33342 (1 *μ*g/ml) and propidium iodide (5 *μ*g/ml) were added to the medium and incubated for 15 min. Both floating and attached cells were collected for the necrosis analysis. The cell pellets were washed with PBS and suspended in PBS. The cell suspension (0.1 ml, ~10^5^ cells) was centrifuged onto glass slides in a cytospinner for 3 min at 300×*g*. The slides were washed with PBS once and fixed in pre-chilled methanol/acetone (3 : 1) for 20 min at −20 °C. The slides were washed with PBS and mounted in Antifade solution. Necrotic cells were examined by fluorescence microscopy at 340/425 nm (Hoechst 33342) and 580/630 nm (propidium iodide).

### RNA sequencing

RNA samples were prepared by RNeasy min kit (Qiagen); a mixture of three biological RNA replicates was used to represent each condition (parental, senescent, revertant). RNA-seq analysis, including rRNA depletion, library preparation, multiplexing and cluster generation, sequencing on Illumina HiSeq2500, and differential gene expression analysis, was performed by Genewiz (South Plainfield, NJ, USA). Gene function analysis was performed using DAVID (Database for Annotation, Visualization and Integrated Discovery, https://david.ncifcrf.gov/) and PANTHER (Protein Analysis THrough Evolutionary Relationships, http://pantherdb.org) programs.

### RNA-seq genes validation

To validate the activated genes identified by RNA-seq, a panel of primers were designed using the Primer3 (v. 0.4.0) software and synthesized by Integrated DNA Technologies (IDT, Coralville, IA, USA). The sequences of primers are listed in Supplementary Table S5. The RNA samples were prepared by RNeasy min kit (Qiagen). High capacity cDNA reverse transcription kit (Appliedbiosystems by Thermo Fisher Scientific, Waltham, MA, USA) was used to transcribe RNA to cDNA. The PowerUp SYBR green master mix kit was employed for the qPCR analysis.

### Flow cytometry

A549 cells were infected with LV-GFP or LV-RFP lentivirus to generate the A549-GFP and A549-RFP cells. Despite the absence of selection markers on the viral vectors, the GFP/RFP labels were stably expressed through multiple generations. The infected cells were treated with 100 nM doxorubicin for 5 days to induce senescence, followed by culture in drug-free medium to generate revertant colonies. The pooled revertant colonies (RFP or GFP labeled) were mixed with the parental cells (GFP or RFP labeled) at 1 : 1 ratio, cultured for eight passages and subjected to FACS analysis using LSRII flow cytometer (BD BioSciences, San Jose, CA, USA). GFP or RFP-parental and RFP or GFP-revertants were mixed 1 : 1 and treated with chemotherapy drugs including 50 *μ*M 5-FU, 40 *μ*M etoposide, 0.5 *μ*M CPT, and 20 *μ*M of cisplatin. The cells were treated for 7 days with the drugs, followed by 2 weeks culture in drug-free medium. The treatment was repeated once and the surviving cells were analyzed by FACS. The data were analyzed using FlowJo software (TreeStar, Ashland, OR, USA).

### *In vivo* tumor growth and metastasis assay

Experimental procedures involving animals were reviewed and approved by the Institutional Animal Care and USE committee of the University of South Florida. Athymic-NCr-nu/nu female mice at 6 weeks (Charles River) were inoculated subcutaneously on both flanks with 2×10^6^ cells containing mixed GFP (parental) and RFP (revertants) or RFP (parental) and GFP (revertants) at 1 : 1 ratio. When the tumor volume reached ~0.3 cm^3^, the tumors were isolated, treated with collagenase D protease (Sigma) to generate single-cell suspension, and analyzed by FACS.

## Publisher’s note

Springer Nature remains neutral with regard to jurisdictional claims in published maps and institutional affiliations.

## Figures and Tables

**Table 1:** Validation of up-regulated genes by RT-qPCR

	*Expression in A549*				
	*Parental (fold)*	*Senescent RNA-seq (fold)*	*Senescent RT-PCR (fold)*	*Revertant RNA-seq (fold)*	*Revertant RT-PCR (fold)*
NPTX1	1	23	40	4	6
IGF2	1	13	12	4.1	3.5
HAPLN3	1	10	38	2.8	7.4
CHAC1	1	25	22	4.2	2.7
BST2	1	207	62	10	1.5
C15orf48	1	207	9	10	2.5
NIPAL4	1	64	25	4.2	2.2
AGT	1	185	98	7.4	3
SPATA18	1	10	19	1.8	2
PIK3R2	1	6	9	1.9	3.6
MT1X	1	9	9	1.9	1.8
PAQR7	1	7	6	2.3	1.7
GJA1	1	5	10	2.4	3.2
LAPTM5	1	26	168	2.4	3.7
SP5	1	16	30	2.4	2.6
COL20A1	1	11	16	2.4	2.4
NAP1L3	1	43	48	2.5	2
FBXL21	1	28	14	2.5	3.5
Tmem26	1	88	28	2.5	3.3
CST1	1	7	11	2.8	4
SNAI2	1	25	74	2.8	5.3
TEN1	1	5	2	2.9	1.7
DYDC2	1	15	9	3	2
ZNF841	1	15	5	3	1.6
AMOT	1	7	28	3	6.2
BARX1	1	13	15	3	3.2
ABCA8	1	6	8	3	3.2
AIM1L	1	10	12	3.2	2.5
CDRT4	1	6	3	3.2	1.3
SLC2A3	1	18	14	3.3	2.5
CPE	1	5	7	3.6	4.4
TRO145	1	30	19	3.9	1.4
HOXD10	1	11	4	5	1.9
PIP	1	53	36	5.6	1.1
EMB	1	5	6	6.5	4.9
SERPINB4	1	26	2	7.9	1.6
QPRT3	1	119	24	20	2.7
SLC7A4	1	714	4	340	1
IFI44	1	395	4324	3	18

**Table 2:** Revertant-activated genes expression in different cell lines

*Gene symbol*	*Gene product*	*Gene function*	*A549*		*H1299*		*MCF7*	
			*Sen/P*	*Rev/P*	*Sen/P*	*Rev/P*	*Sen/P*	*Rev/P*
SP5	Transcription factor	Invasion	30	5	7	0.5	1.4	3
**NAP1L3**	**Nucleosome assembly**	**Metastasis**	**47**	**2**	**30**	**5**	2	0.1
IGF2	Growth factor	Metastasis	12	3	NS	NS	2.7	1
SPATA18	Mitochondria protein	Spermatogenesis	20	2	NS	NS	9.5	1
**IFI44**	**Interferon-induced protein**	**Recurrence**	**400**	**17**	**110**	**2**	9.6	1
HAPLN3	Proteoglycan link protein	Adhesion	38	7	NS	NS	2.5	1.4
**AGT**	**Angiotensinogen**	**Metastasis**	**100**	**3**	**270**	**28**	**10.9**	**3.3**
TMEM26	Trans membrane protein	Recurrence	28	3	NS	NS	1.7	0.7
AMOT	Angiomotin	Metastasis	27	10	3	0.3	0.7	1.8
**CPE**	**Carboxypeptidase E**	**Metastasis**	**7**	**4**	**12**	**3**	1.7	1.3
**EMB**	**Embigin**	**Metastasis**	**6**	**4**	**5**	**1.5**	2.3	1
CST1	Cystatin	Invasion	11	10	NS	NS	**2.6**	**48**
CST4	Cystatin	Invasion	16	10	NS	NS	**27.9**	**3.2**
**MAFB**	**Oncogene**	**Oncogene**	**250**	**6**	**90**	**10**	3.9	0.7
**PRSS22**	**Protease**	**Motility**	**30**	**3**	**400**	**11**	**6.5**	**1.8**
**ACKR3**	**Chemokine receptor**	**Metastasis**	**250**	**3**	**25**	**11**	3.9	0.9
CDH6	Cadherin	Metastasis	100	5	NS	NS	NS	NS
SYK	Tyrosine kinase	Inhibit motility	14	8	NS	NS	1	1.6
GBP1	Guanylate binding protein	Metastasis	100	11	NS	NS	**3.2**	**1.6**

Note: Bolded entries highlight genes that are activated in both A549 and H1299 revertants.

NS, no signal; P, parental cells; Rev, revertants; Sen, senescent cells.
