# Supplementary material for: Tumor cell senescence response produces aggressive variants
Source: Cell Death Discov. 2017 Aug 21;3:17049–. doi: 10.1038/cddiscovery.2017.49 (PMC5563524; doi:10.1038/cddiscovery.2017.49)
Supplement: Supplementary Figures [file cddiscovery201749-s1.docx]

**Supplemental figures and legend**

**Figure S1. Stress treatments stimulate senescence reversal.** (a) A549 cells were treated with 300 nM CX5461 for 7 days to induce senescence, followed by treatment with 2 μM ABT-737, 500 nM camptothecin, and glucose deprivation. The cells were then washed and cultured in drug-free media for 3-6 weeks. Colonies were stained with crystal violent. Bar = 20 µm.

**Figure S2. Depletion of Bcl2 in senescent cells promotes cell cycle re-entry.** (a, b) Senescent A549 cells were transfected with 50 nM control siRNA or Bcl2 siRNAs and then cultured in regular medium until colony formed. Colonies were photographed and stained using crystal violet. Bar = 20 µm. (c) Knockdown of Bcl2 by siRNA was confirmed by RT-qPCR. Values are mean ± SD of triplicates. (d) Bcl2 knockdown and induction of PARP cleavage by siRNA were confirmed by western blot. (e) Senescent H460 cells induced by doxorubicin were treated with 5 μM ABT-737 for 5 days, followed by culture in drug-free medium for 21 days and stained for colony formation by crystal violet.

**Figure S3. Induction of necrosis stimulates senescence reversal.** (a) Senescent A549 cells were treated with pan-caspase inhibitor QVD-OPh and topoisomerase I inhibitor CPT for 48 hrs and photographed for the induction of cell death. Bar = 20 µm. (b) Senescent A549 cells were treated with 500 nM CPT or cultured in glucose-free medium for 2 days. The cells were incubated with Hoechst 33342 and propidium iodide, and photographed for staining of nuclear DNA. The staining of nuclear DNA by propidium iodide was used as an indicator of membrane leakage and necrotic cell death. Bar = 10 µm.

**Figure S4. Senescence revertants do not have increased expression of most cancer stem cell markers.** The expression levels of a panel of cancer stem cell markers were analyzed by RT-PCR in cycling parental A549, senescent A549, and senescence revertants. The changes relative to parental A549 are shown in the heat map.
