# Supplementary material for: Tumor cell senescence response produces aggressive variants
Source: Cell Death Discov. 2017 Aug 21;3:17049–. doi: 10.1038/cddiscovery.2017.49 (PMC5563524; doi:10.1038/cddiscovery.2017.49)
Supplement: Supplementary Table S5 [file cddiscovery201749-s6.docx]

**Table S5**

| Gene | Forward primer | Reverse primer |
| --- | --- | --- |
| NPTX1 | accgaggagagggtcaagat | caggctcttcttcaccttgg |
| IGF2 | acaccctccagttcgtctgt | ggggtatctggggaagttgt |
| HAPLN3 | ctcagcaggatgcagtgtgt | tagaagggcagtccgtagga |
| CHAC1 | ctcaagcgctgtggattttc | gatcttcaaggagcgtcacc |
| BST2 | tgctggggataggaattctg | tcagctcttgttgcaggaga |
| C15orf48 | agcctcatctttcgctgtgt | tggtcaccctttggacattt |
| NIPAL4 | gggagagtctgaacctgctg | caaacacgatgaaccctgtg |
| AGT | ccacttccaagggaagatga | agaggcatagtgaggctgga |
| SPATA18 | gcagctacgactcggatttc | aggactccgacttcgagaca |
| PIK3R2 | cctggcacctatgtggagtt | acatcaggtggggagaactg |
| MT1X | gttggctcctgtgcctgt | cacagcagctgcacttgtct |
| PAQR7 | tcagacctggcgcttctatt | aggtgaaagaggcaaggaca |
| GJA1 | atgagcagtctgcctttcgt | tctgcttcaagtgcatgtcc |
| LAPTM5 | gagcctactgatcggcgtag | aggtgggcacttccatgtag |
| SP5 | caaggtgtacgggaagacgt | gtgagtcttgacgtgcttcg |
| COL20A1 | gagacacctgggcttctcag | cacggacagtgtaggtggtg |
| NAP1L3 | ggggatgggaaattgaagat | cttctcgtggttccagcttc |
| FBXL21 | tgtccagttgtcaggagcag | gcacaggcccgatctagtaa |
| Tmem26 | tgctgcagtttccacttgac | acaccaccacgaggaagttc |
| CST1 | acaaggccaccaaagatgac | tcaccagggaccttctgttc |
| SNAI2 | ctggttgcttcaaggacaca | ttggagcagtttttgcactg |
| TEN1 | ttagtgcaggccaagttcct | gagcctctgtcctgctgatg |
| DYDC2 | gacacaaacccccttgagaa | tcatgctgaaaagcctcctt |
| ZNF841 | ccatgcaggaaagaaaccat | gcaagacgtgaacgttgaaa |
| AMOT | tctgctcctgctcagactca | acaggcccatctgttttgtc |
| BARX1 | aacgcttcgagaagcagaag | ctcgctcgttggaattgagt |
| ABCA8 | caggaccagctgaagtctcc | tcagcctctgccatgtagtg |
| AIM1L | ctggctccaagaacttccag | ctcagtcctggccatagctc |
| CDRT4 | gggcatcaaggcagaataaa | agtgtgtgggttctgggatc |
| SLC2A3 | accggcttcctcattacctt | aggctcgatgctgttcatct |
| CPE | acatcatgccttccctgaac | gacagccttggtctcaggag |
| TRO145 | gcaactgaaagccagactcc | ctcatccccattcagatgct |
| HOXD10 | gctccttcaccaccaacatt | aaatatccagggacgggaac |
| PIP | tactgcctgcctatgtgacg | aaaccggttgtttttgatgg |
| EMB | cccagattcgccttttacaa | ctgcattcaaatccccagat |
| SERPINB4 | tcagtgaagccaacaccaag | tgttgcagctttttctgtgg |
| QPRT3 | tggagaagtatgggctcctg | ttccaccttcagagcgaagt |
| SLC7A4 | catccgcaacttcactgaga | agcccaggatgacaatgaag |
| IFI44 | ttcgatgcgaagattcactg | cccttggaaaacagacctca |
| CDH6 | accacaataggctccgtcac | tgccatagcagtgtttctcg |
| CST4 | gccatcagcgagtacaacaa | gaaggcacaggtgtccaagt |
| MAFB | tgctcaagttcgacgtgaag | cgccatccagtacagatcct |
| PRSS22 | tctgcctacctgatgcctct | ccagtacagatggctgcaga |
| ACKR3 | acgtggtggtcttccttgtc | aaggccttcatcagctcgta |
| SYK | cgtatgagccagaacttgca | gccacggttttcacaacttt |
| GBP1 | agaagtgaaggcgggaattt | ctcagctttcacacgttcca |
| GAPDH | gagtcaacggatttggtcgt | gacaagcttcccgttctcag |
